# Sex Difference in Self-Reported Sexual Functioning Among Frail Older Adults

**DOI:** 10.1093/geroni/igaa057.1002

**Published:** 2020-12-16

**Authors:** Brianne Olivieri-Mui, Sandra Shi, Ellen McCarthy, Dae Kim

**Affiliations:** 1 Hebrew SeniorLife, Harvard Medical School, Roslindale, Massachusetts, United States; 2 Beth Israel Deaconess Medical Center, Boston, Massachusetts, United States; 3 Hebrew SeniorLife, Harvard Medical School, roslindale, Massachusetts, United States

## Abstract

Frailty may differentially impact how older adult males and females perceive sexual functioning, an important part of well-being. We assessed the level of frailty (robust, pre-frail, frail) for anyone with data on 11 sexual functioning questions asked in wave 2 of the National Social Life, Health, and Aging Project, 2010-2011 (n=2060). Questions covered five domains: overall sexual function (OSF), sexual function anxiety (SFA), changes in sexual function (CSF), erectile/vaginal dysfunction (EVD), and masturbation. Logistic regression identified sex differences in frailty and reporting worse sexual functioning. Linear regression predicted the number of domains reported as worse. Among males (n=1057), pre-frailty meant higher odds of reporting SFA (OR 1.8 95%CI 1.2-6.6), CSF (OR 1.7 95%CI 1.1-2.7), and EVD (OR 1.5 95%CI 1.0-2.2). Among females (n=1003), there was no difference in reporting by frailty. Females were more likely to report worse OSF (Robust: OR 7.4, 95%CI 4.8-11.4; Pre-frail: OR 6.2, 95%CI 3.9-9.9; Frail: OR 3.4 95%CI 1.7-6.6), but less likely to report SFA (Robust OR .3, 95%CI .2-.5; Pre-frail OR .2, 95%CI .1-.3; Frail OR .2 95%CI .1-.3). Pre-frail and frail females reported fewer domains as worse (Pre-frail coefficient -0.21 SE 0.09, Frail -0.43 SE 0.14). As frailty worsened, males reported more domains as worse (Pre-frail 0.24 SE 0.07, Frail 0.29 SE 0.08). Self-reported sexual functioning differs by sex at all levels of frailty, and reporting by males, but not females, changes with frailty. Providers should be aware that sexual functioning is of importance to both sexes despite varying degrees of frailty.

